# Conformational Analysis of *N*-Alkyl-*N*-[2-(diphenylphosphoryl)ethyl]amides of Diphenylphosphorylacetic Acid: Dipole Moments, IR Spectroscopy, DFT Study

**DOI:** 10.3390/molecules26164832

**Published:** 2021-08-10

**Authors:** Anastasiia Kuznetsova, Denis Chachkov, Oleg Artyushin, Natalia Bondarenko, Yana Vereshchagina

**Affiliations:** 1 Department of Physical Chemistry, A.M. Butlerov Institute of Chemistry, Kazan Federal University, Kremlevskaya 18, 420008 Kazan, Russia; kuznetsovaanastan@gmail.com; 2 Kazan Department of Joint Supercomputer Center of Russian Academy of Sciences, Branch of Federal Scientific Center “Scientific Research Institute for System Analysis of the RAS”, Lobachevskogo 2/31, 420111 Kazan, Russia; de2005c@gmail.com; 3 A.N. Nesmeyanov Institute of Organoelement Compounds, Russian Academy of Sciences, Vavilova 28, 119991 Moscow, Russia; oleg.artyushin@gmail.com; 4 Institute of Chemical Reagents and High Purity Chemical Substances of NRC “Kurchatov Institute”, Bogorodsky val 3, 107076 Moscow, Russia; bond039@mail.ru

**Keywords:** carbamoylphosphine oxides, phosphorylated acetamides, dipole moments, conformational analysis, DFT calculations

## Abstract

Experimental and theoretical conformational analysis of *N*-methyl-*N*-[2-(diphenylphosphoryl)ethyl]diphenylphosphorylacetamide, *N*-butyl-*N*-[2-(diphenylphosphoryl)ethyl]diphenylphosphorylacetamide, and *N*-octyl-*N*-[2-(diphenylphosphoryl)ethyl]diphenylphosphorylacetamide was carried out by the methods of dipole moments, IR spectroscopy, and Density Functional Theory (DFT) B3PW91/6-311++G(df,p) calculations. In solution, these *N*,*N*-dialkyl substituted bisphosphorylated acetamides exist as a conformational equilibrium of several forms divided into two groups—with *Z*- or *E*-configuration of the carbonyl group and alkyl substituent, and *syn* or *anti* arrangement of the phosphoryl-containing fragments relative to the amide plane. The substituents at the phosphorus atoms have eclipsed *cis*- or staggered *gauche*-orientation relative to the P=O groups, and *cis* orientation of the substituents is due to the presence of intramolecular H-contacts P=O...H−C_phenyl_ or p,π conjugation between the phosphoryl group and the phenyl ring. Preferred conformers of acetamides molecules are additionally stabilized by various intramolecular hydrogen contacts with the participation of oxygen atoms of the P=O or C=O groups and hydrogen atoms of the methylene and ethylene bridges, alkyl substituents, and phenyl rings. However, steric factors, such as a flat amide fragment, the bulky phenyl groups, and the configuration of alkyl bridges, make a significant contribution to the realization of preferred conformers.

## 1. Introduction

Derivatives of carbamoylmethylphosphine oxides (CMPO) are known as effective extractants of rare earth elements and actinides from mineral acid solutions [1,2,3,4,5,6,7,8,9,10,11,12,13,14,15,16,17]. The high coordinating ability of these compounds is due to the presence of polar phosphoryl and amide groups. CMPO are used in industrial processes for reprocessing spent nuclear fuel [18,19] and for the processing of radioactive waste and the separation of transplutonium elements [20,21], as well as for the preparation of composite materials for column chromatography [22,23,24]. Carbamoylmethylphosphine oxides modified with a dialkylamidomethyl coordination group in the methylene bridge are potential neuroprotective agents [25,26]. Manganese (II) complexes with CMPO ligands exhibit luminescent properties [27]. Palladium complexes containing phosphoryl-substituted CMPO as ligand demonstrate catalytic activity in Suzuki reaction [28].

Although carbamoylmethylphosphine oxides have found active practical application for a long time, their spatial structure has been poorly studied. The literature contains data on the structure of complexes of CMPO with rare earth elements and actinides [17,29,30], manganese (II) [27], and mercury (II) [31] in the solid state. The crystal structures of (diethylcarbamoyl)methyldiphenylphosphine oxide and its protonated form [32], diphenylmorpholine carbamoylmethyl phosphine oxide [33], diphenyl-*N*,*N*-dimethylcarbamoylmethylphosphine oxide [34], *N*-aryl-substituted CMPO modified with a phosphoryl group, and its complexes with Pd (II) and Re (I) [35] were determined by X-ray diffraction. Information on the structure in solution is available only for P(X)-modified (X = O, S) *N*-aryl-substituted CMPOs: experimental and theoretical conformational analysis by the methods of dipole moments and quantum chemistry showed that in solution, these compounds exist as an equilibrium of several conformers with intramolecular hydrogen bonds H⋯X [36]. The deficiency of data on the CMPO’s conformational behavior is a problem in describing the coordinating properties of such compounds and explaining the reaction mechanisms with their participation. *N*-Alkyl-*N*-[2-(diphenylphosphoryl)ethyl]amides of diphenylphosphorylacetic acid were synthesized recently [37], but their polarities and conformational structures have not been studied.

**Scheme 1:**
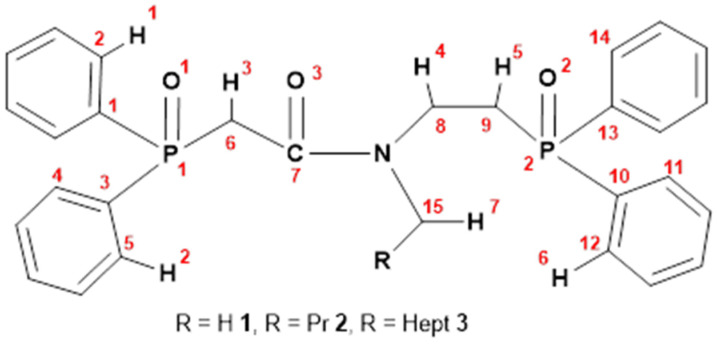
Structures of compounds **1**–**3**, selected atom numbers are given.

In the present work, we investigated the spatial structure of *N*-methyl-*N*-[2-(diphenylphosphoryl)ethyl]diphenylphosphorylacetamide **1**, *N*-butyl-*N*-[2-(diphenylphosphoryl)ethyl]diphenylphosphorylacetamide **2**, and *N*-octyl-*N*-[2-(diphenylphosphoryl)ethyl]diphenylphosphorylacetamide **3** (Scheme 1) by the methods of dipole moments, IR spectroscopy, and quantum chemistry DFT B3PW91/6-311++G(df,p).

## 2. Results and Discussion

### 2.1. Methodology

The studied amides of phosphorylacetic acid (phosphorylacetamides) **1**–**3** are polyfunctional polar compounds. Two phosphoryl groups with bulky phenyl substituents at the phosphorus atom are linked through alkyl bridges by an amide group; there is also a flexible alkyl substituent at the nitrogen atom. All fragments of a molecule can freely rotate about single bonds, and functional groups can participate in intra- and intermolecular non-covalent interactions, which leads to the emergence of a large number of possible conformers.

The method of dipole moments is a sensitive instrument for the determination of structure and the study of fine features of spatial and electronic structure of polar organic and organoelement compounds in solution. To determine the experimental values of the dipole moments, we used the second Debye method based on the measurement of the dielectric permittivity of the dilute solutions of the polar substance in a nonpolar solvent [38].

To search the possible conformations of isolated molecules of **1**–**3**, we applied the Density Functional Theory (DFT) with B3PW91 hybrid functional. This method has been successfully used to study the polarity and spatial structure of similar organophosphorus compounds with double bond phosphorus–chalcogen and aryl substituents [36,39]. The choice of the B3PW91 method was also based on the data [40].

### 2.2. Polarity of Phosphorylacetamides ***1***–***3***

We have determined previously unknown polarities of compounds **1**–**3**. The experimental values of the dipole moments were determined in benzene solutions using the second Debye method based on the measurement of the dielectric constant of the dilute solutions of a polar substance in a nonpolar solvent. The experimental dipole moments of **1**–**3** are listed in Table 1; their values are sufficiently high (Table 1) and are typical for the polarities of the compounds of tetra-coordinated phosphorus (2.5–5.0 Debye) [41].

### 2.3. Experimental and Theoretical Conformational Analysis of Phosphorylacetamides ***1***–***3***

Conformers for the compounds were built using the Gauss View 6.0 imaging software. All possible conformations for compounds **1**–**3** were built by sequential rotation of the parts of the molecules relative to single bonds. Conformations with overlapping atoms or with a too close arrangement, which does not correspond to the physical meaning, were immediately discarded. Thus, there were about a thousand conformers. First, the calculations were carried out for them using small basis set 6-31G(d). Based on these results, we have discarded degenerate structures. Then, conformers with high relative energies (more than 15 kJ/mol) were discarded as unlikely. At the next stage, the calculations were carried out using the extended basis set 6-311++G(df,p). We considered the preferred conformers with relative energies less than 6 kJ/mol without taking into account the mirror isomers with identical energies and theoretical dipole moments. For all preferred conformers, their relative energies and theoretical polarities were computed, and the dipole moments were calculated according to the vector-additive scheme (Table 2). Conformational diversity of the compounds **1**–**3** is due to the presence of a large number of rotation axes and several functional groups in their molecules. The percentage of conformers in the mixture was calculated on the basis of the theoretical values of Gibbs energies (Table 2).

**Figure 1:**
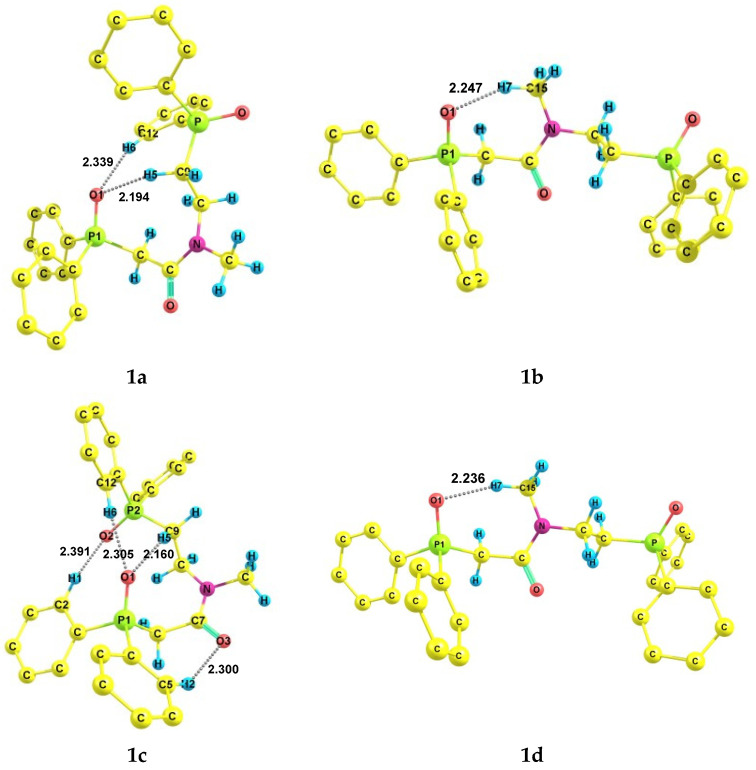

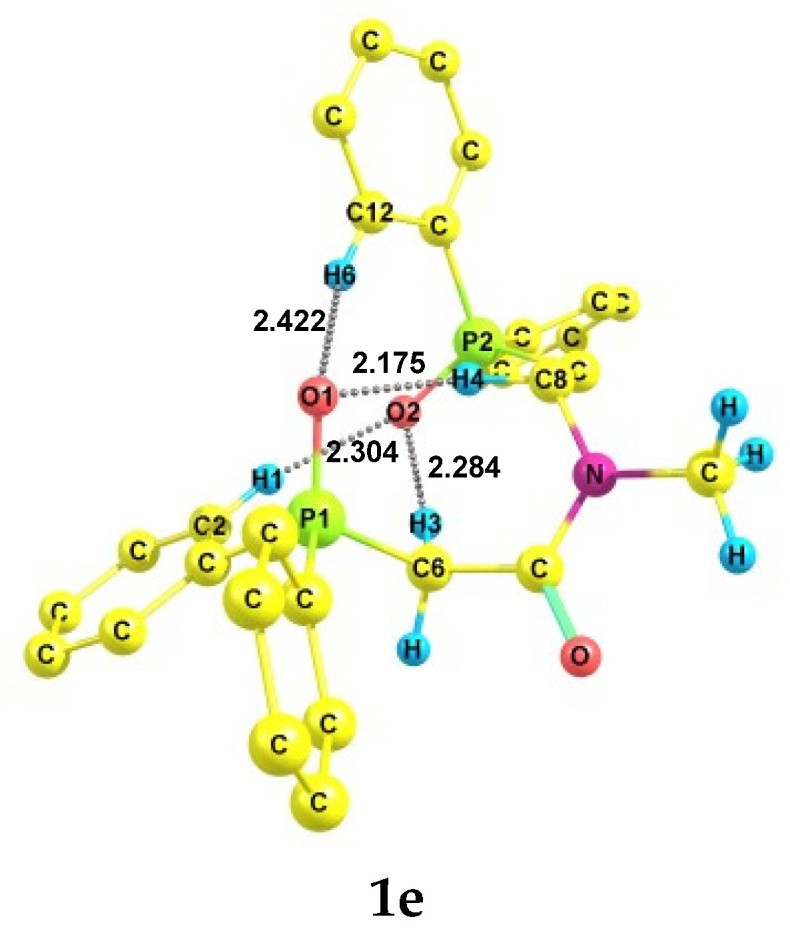
Preferred conformers of **1** according to DFT calculations (some hydrogen atoms are omitted). Distances are given in Å.

According to the results of theoretical calculations, five energetically preferred conformers were found for *N*-methylacetamide **1** (Figure 1); their characteristics are listed in Table 2 and Table 3. In conformers **1a**–**1e**, the phosphorus atoms have a pyramidal structure, the phenyl substituents have *cis*- or *gauche*-orientation, and the methyl or ethyl bridges have *gauche*-orientation relative to the P=O bonds (*α*, β, *γ*, ι, κ, and λ angles in Table 3).

The amide fragment is flat, the carbonyl and methyl groups have *Z*-orientation relative to the C_sp2_–N bond in conformers **1a**, **1c**, **1e**, and *E*-orientation in **1b** and **1d**; the bulky diphenylphosphoryl fragments are *syn*-located relative to amide plane in conformers **1a**–**1c** and *anti*-located in **1d** and **1e** (Figure 1).

The conformers **1a**–**1e** differ in the arrangement of the following bonds relative to each other: the P1–C_sp3_ and C=O bonds have synclinal orientation in **1c** and anticlinal orientation in all other conformers; the N–C_sp3_ and C_sp3_–P2 bonds are practically coplanar in conformers **1a**–**1d** (antiperiplanar orientation), and they have anticlinal orientation in **1e**. The C_sp3_–C_sp3_ and N–C_sp3_(methyl) bonds have synclinal orientation in conformers **1a**, **1c**, **1e**, and anticlinal orientation in **1b** and **1d**; that is, the ethyl fragment is practically perpendicular to the amide plane (Table 3). The N–C_sp3_ and C_sp3_–P2 bonds lie in the same plane in **1a**–**1e** (θ angle, Table 3).

All conformers **1a**–**e** are additionally stabilized by intramolecular hydrogen contacts (Figure 1, Table 4). In conformers **1b** and **1d**, there is an interaction between the oxygen atom of the phosphoryl group P1=O and the hydrogen atom of the methyl substituent, resulting in the formation of seven-membered intramolecular heterocycles. The bifurcate hydrogen bond between the oxygen atom of the P1=O group and the hydrogen atoms of the ethylene bridge and one of the phenyl rings at the second diphenylphosphoryl group is observed in **1a**, **1c**, and **1e**, resulting in the formation of seven- or eight-membered intramolecular cycles. In addition, in conformer **1c**, H-contacts between the oxygen atom of the second phosphoryl group P2=O and the hydrogen atom of one of the phenyl substituents at the P1 atom (C2–H1⋯O2=P 2 2.391 Å, C2–H1⋯O2 156°), as well as between the oxygen atom of the carbonyl group and the hydrogen atom of the other phenyl substituent at the P1 atom (C5–H2⋯O3–C7 2.300 Å, C5–H2⋯O3 141°) are observed. The second bifurcate bond is formed by the oxygen atom of the P2 = O group and hydrogen atoms of the phenyl ring at the first phosphorus atom and the methylene bridge (C2–H1⋯O2 = P2 2.304 Å, C2–H1⋯O2 167° and C6–H3⋯O2=P2 2.283 Å, C6–H3⋯O2 164° respectively) in conformer **1e**.

**Figure 2:**
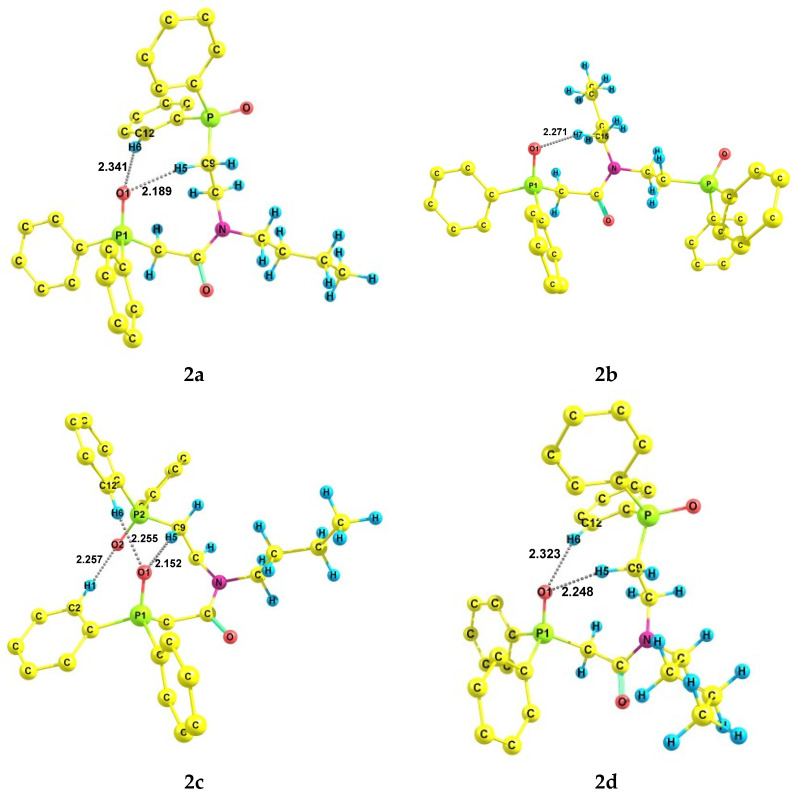

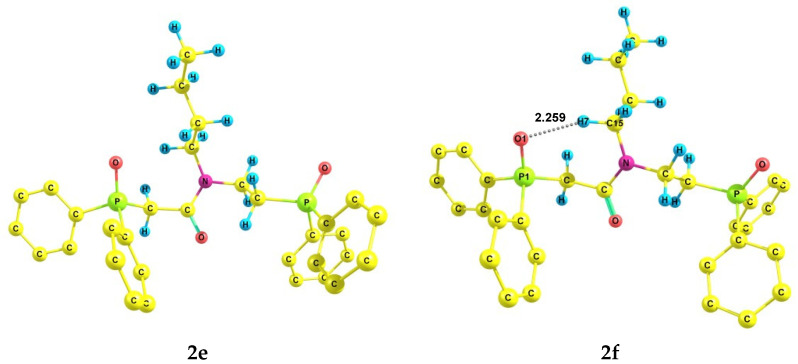
Preferred conformers of **2** according to DFT calculations (some hydrogen atoms are omitted). Distances are given in Å.

Elongation of the alkyl substituent at the nitrogen atom led to an increase in the number of energetically preferred conformers of *N*-butyl-*N*-[(2-(diphenylphosphoryl)ethyl]diphenylphosphorylacetamide **2** to 6 (Figure 2); their characteristics are listed in Table 2 and Table 3. The common features of conformers **2a**–**f** are a pyramidal structure of phosphorus atoms and a planar amide fragment, which are also characteristic for conformers **1a**–**e**. In conformers **2a**–**f**, the phenyl substituents are predominantly *cis*-oriented, and the methyl or ethyl bridges are *gauche*-orientated relative to the P = O bonds (*α*, β, *γ*, ι, κ, and λ angles in Table 3).

The carbonyl group and butyl substituent at the nitrogen atom have mutual *Z*-orientation in conformers **2a**, **2c**, **2d**, and *E*-orientation in **2b**, **2e**, and **2f** (*ε* angle, Table 3); the bulky diphenylphosphoryl fragments are *syn*-located relative to amide plane in conformers **2a**–**e** and *anti*-located in **2f** (Figure 2).

The difference between conformers is due to the different mutual arrangement of the following bonds: P1–C_sp3_ and C_sp2_=O bonds have synclinal orientation in **2c** and anticlinal orientation in all other forms (δ angle, Table 3). The C_sp3_–C_sp3_ and N–C_sp3_(butyl) bonds have synclinal orientation in **2a** and **2d**, and anticlinal orientation in **2b**, **2c**, **2e**, and **2f** (η angle, Table 3); the N–C_sp3_ and C_sp3_–P2 bonds are antiperiplanar in **2a**–**2f** (θ angle, Table 3).

An additional variety of conformers **2a**–**f** compared to compound **1** is due to the presence of a longer alkyl substituent. However, the difference between preferred conformers is only due to the rotation relative to the N–C_sp3_(R) bond, because conformers with a zigzag alkyl radical possess low energies. The C_sp2_–N and C_sp3_(R)–C_sp3_(R) bonds have synclinal orientation in **2a**, **2c**, and **2d** and anticlinal orientation in **2b**, **2e**, and **2f** (ζ angle, Table 3).

For conformers **2a**–**d** and **2f**, an additional stabilization effect is observed due to the formation of intramolecular hydrogen contacts (Table 4). In **2b** and **2f**, the interaction occurs between the oxygen atom of the group P1=O and one of the hydrogen atoms of the butyl substituent, resulting in the formation of seven-membered intramolecular rings. In conformers **2a**, **2c**, **2d**, and **2f**, a bifurcate hydrogen bond is observed between the oxygen atom of the group P1=O and the hydrogen atoms of the ethylene bridge and one of the phenyl rings at the P2 atom, resulting in the formation of seven- or eight-membered intramolecular cycles. In addition to the bifurcate bond, a contact between the oxygen atom of the second phosphoryl group and the hydrogen atom of one of the phenyl substituents at the P1 atom is formed in conformer **2c** (C2–H1⋯O2=P2 2.257 Å, C2–H1⋯O2 176°).

**Figure 3:**
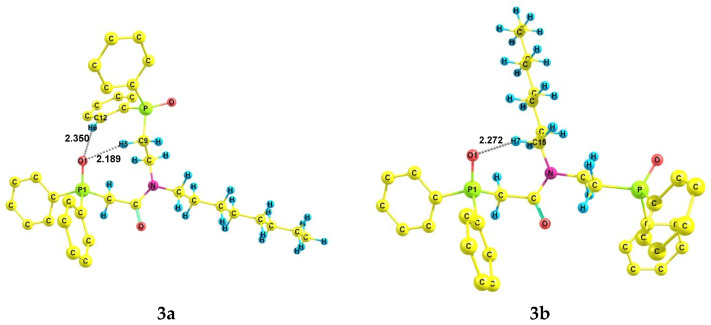

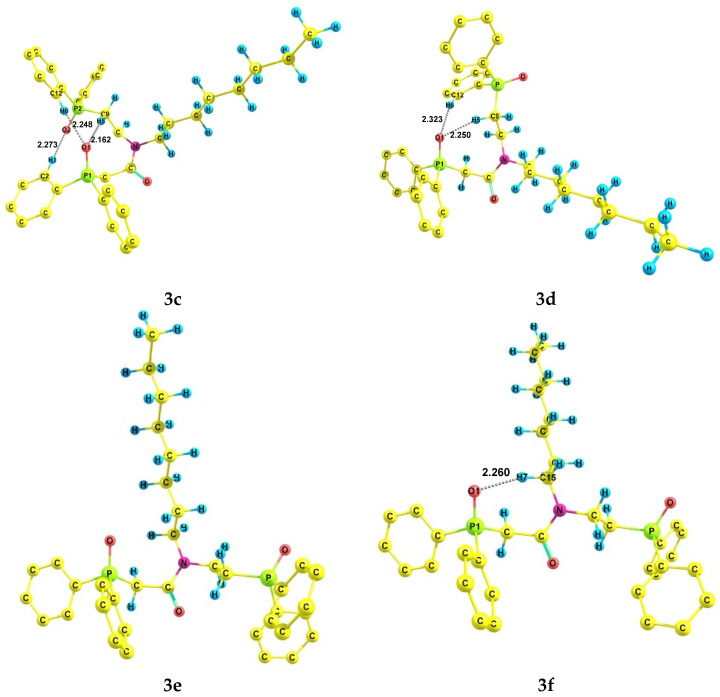
Preferred conformers of **3** according to DFT calculations (some hydrogen atoms are omitted). Distances are given in Å.

According to the quantum chemical calculations, for *N*-octyl-*N*-[(2-(diphenylphosphoryl)ethyl]diphenylphosphorylacetamide **3**, six energetically preferred conformers were found (Figure 3); their characteristics are listed in Table 2 and Table 3. As for compounds **1** and **2**, conformers **3a**–**f** are characterized by a pyramidal structure of the phosphorus atoms and a flat structure of the nitrogen atom. In conformers **3a**–**f**, the phenyl substituents are predominantly *cis*-oriented and the methyl or ethyl bridges are *gauche*-orientated relative to the P=O groups (*α*, β, *γ*, ι, κ, and λ angles in Table 3).

As in the case of butyl substituted amide **2**, the carbonyl group and octyl substituent at the nitrogen atom have mutual *Z*-configuration in conformers **3a**, **3c**, **3d**, and *E*-configuration in **3b**, **3e**, and **3f** (dihedral angle *ε*, Table 3); the diphenylphosphoryl fragments are *syn*-located relative to amide plane in conformers **3a**–**e** and *anti*-located in **3f** (Figure 2).

Conformers **3a**–**e** differ in the structure of the carbamoylmethyl fragment and the ethylene bridge; the P1–C_sp3_ and C=O bonds are synclinal in **3c**, while in the rest of the conformers, these bonds are anticlinal (dihedral angle δ, Table 3). The C_sp3_–C_sp3_ and N–C_sp3_(R) bonds are synclinal in **3a** and **3d**, and anticlinal in **3b**, **3c**, **3e**, and **3f** (dihedral angle η, Table 3). The N–C_sp3_ and C_sp3_–P2 bonds have antiperiplanar mutual arrangement in conformers **3a**–**f** (dihedral angle θ, Table 3).

An additional difference between conformers is due to the rotation of the octyl substituent relative to the N–C_sp3_(R) bond. The C_sp2_–N and C_sp3_(R)–C_sp3_(R) bonds have mutual synclinal arrangement in **3a**, **3c**, and **3d** and anticlinal arrangement in **3b**, **3e**, and **3f** (dihedral angle ζ Table 3).

The intramolecular hydrogen contacts are possible in conformers **3a**–**3d** and **3f** (Table 4). In conformers **3b** and **3f**, an interaction between the oxygen atom of the P1=O group and one of the hydrogen atoms of the octyl substituent is observed, resulting in the formation of seven-membered intramolecular cycles. The bifurcate hydrogen bonds between the oxygen atom of the first phosphoryl group and the hydrogen atoms of the ethylene bridge and one of the phenyl rings at the P2 atom arise in conformers **3a**, **3c**, **3d**, and **3f**. Moreover, a contact between the oxygen atom of the second phosphoryl group and the hydrogen atom of one of the phenyl substituents at the P1 atom is formed in conformer **3c** (C2–H1⋯O2 = P2 2.273 Å, C2–H1⋯O2 176°).

**Figure 4:**
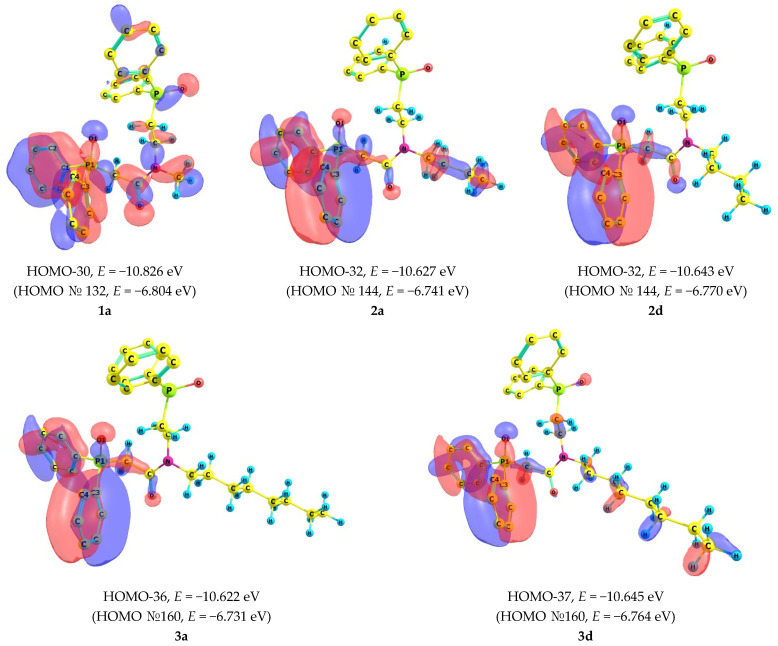
Visualization of the molecular orbitals № 102 for **1a**, № 112 for **2a**, № 112 for **2d**, № 124 for **3a**, and № 123 for **3d** corresponding to the interaction of the P=O bond and phenyl substituents. The positive and negative wave functions are shown as blue and red areas, respectively. The isocontour value is 0.02 a.u. The frontier orbitals are not given, since we do not consider the reactivity of the compounds in this context.

It should be noted that in conformers **1a**, **2a**, and **2d**, **3a**, and **3d**, the phosphoryl group P1=O and one of the phenyl substituents at the P1 atom have a completely eclipsed *cis*-orientation (*α* angle, Table 3); their coplanar arrangement promotes p,π-conjugation in the molecules and additionally stabilizes these conformers (Figure 4 and Figure S1). A similar fact was previously described for (arylcarbamoylmethyl)diphenylphosphine oxides and sulfides [38].

Conformers **1c**, **1e**, **2c**, and **3c** have the smallest dipole moments, both theoretical and calculated by the vector-additive scheme (exaltation between the experimental and calculated values—Δμ is 3.38, 1.75, 2.85, and 2.49 D, respectively—see Table 1 and Table 2), which can presumably be explained by the largest number of intramolecular hydrogen contacts in these forms, causing a decrease in their dipole moments. However, these low-polarity forms have slightly higher Gibbs energies, and their content in conformational equilibrium is insignificant (Table 2).

**Figure 5:**
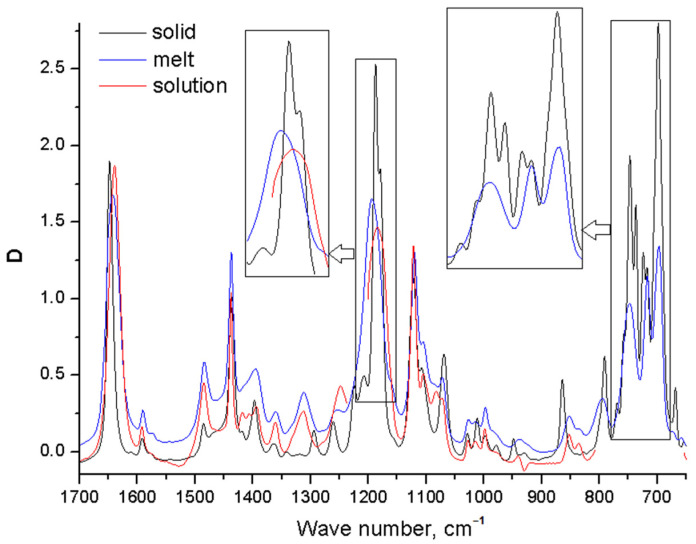
IR spectra of compound **1** in different aggregate states. D is the absorbance.

We have registered the IR spectra of compounds **1**–**3** in the solid state, in the melt, and in solution of the trichloromethane (Figure 5, Figures S2 and S3). The comparison of IR spectra of **1**–**3** showed a change in the number of bands in the range of 700–800 cm^−1^, corresponding to the out-of-plane deformation vibrations of the C–H bonds in the phenyl substituents. For compound **1**, three absorption bands are observed in the melt, whereas seven bands are observed in the solid sample: the 717 cm^−1^ band splits in two (717 cm^−1^ and 724 cm^−1^), and new bands at 737 cm^−1^, 758 cm^−1^, and 770 cm^−1^ appear. In the case of compound **2**, there are also three absorption bands in the melt and seven bands in the solid state: the 717 cm^−1^ band splits in two (715 cm^−1^ and 723 cm^−1^), the 793 cm^−1^ band splits in two (790 cm^−1^ and 795 cm^−1^), and new bands at 740 cm^−1^ and 774 cm^−1^ appear. The IR spectra of **3** also contain three vibrational bands in the melt and six bands in the solid state. New bands at 760 cm^−1^, 777 cm^−1^, and 809 cm^−1^ appear. These IR spectral data indicate the presence of conformational equilibrium in acetamides **1**–**3**.

It should be noted that there is no noticeable shift and no change in the number of vibration bands of the phosphoryl and carbonyl groups when changing the state from solid to liquid (Table 5). A split peak is observed for the stretching vibrations of the P=O bond in the spectrum of the solid sample of **1** as well as in simulated spectra of conformers **1a**–**e**, **2a**–**f**, and **3a**–**f**. The theoretical frequencies of the stretching vibrations of the C=O and P=O groups were calculated using the scaling factor 0.96 [42].

A comparison of experimental and calculated according to vector scheme dipole moments, IR data, and theoretical results showed that in solution, compounds **1**–**3** exist as a conformational equilibrium of several forms, among which highly polar ones prevail.

**Figure 6:**
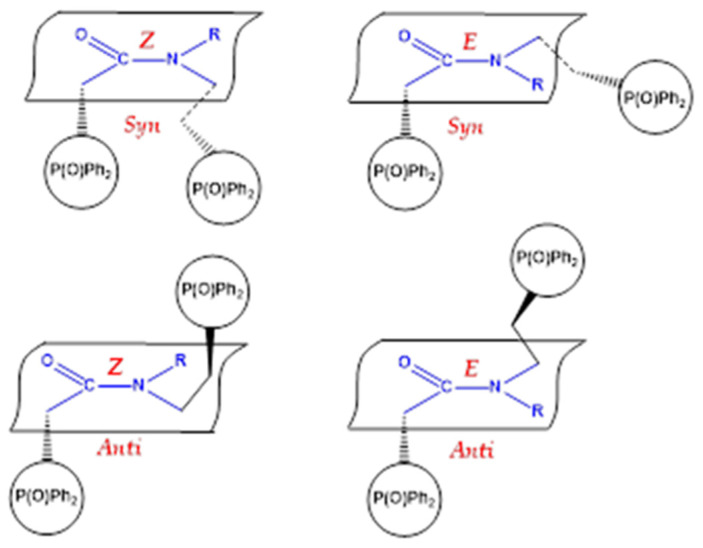
Structures of preferred conformers of **1**–**3**.

Preferred conformers **1a**–**e**, **2a**–**f**, and **3a**–**f** can be divided into two groups, with *Z*- or *E*-configuration of the carbonyl and alkyl groups relative to the C_sp2_–N bond in the planar amide fragment; in addition, the phosphoryl-containing fragments can be *syn*- or *anti*-oriented relative to the amide plane (Table 2, Figure 6).

According to NMR spectroscopy data (^1^H, ^13^C{1H}, ^31^P{1H}, in CDCl_3_ solutions), diphenylphosphinylacetic acid amides **1**–**3** exist as two conformers in a ratio of ≈2.5–3.0:1, depending on the structure of the alkyl substituent at the nitrogen atom [37]. However, it was not possible to establish the structure of these two conformers.

A comparative analysis of the obtained experimental and theoretical results and the data of NMR spectroscopy [37] allowed us to conclude that in solution, compounds **1**–**3** exist as two sets of conformers: in the first set, the C=O and alkyl groups have *Z*-configuration in rotamers **1a**, **1c**, and **1e**; **2a**, **2c**, and **2d**; **3a**, **3c**, and **3d**, while in the second set, these groups have *E*-configuration in rotamers **1b** and **1d**; **2b**, **2e**, and **2d**; **3b**, **3e**, and **3d**, and in all forms except **1e**, **2f**, and **3f**, the phosphoryl-containing fragments are *syn*-located relative to the plane of the amide fragment. We believe that the first set of conformers corresponds to the minor conformer, and the second set corresponds to the majority conformer, found by NMR spectroscopy data, which is confirmed by the total conformer ratio (Table 2). Elongation of the alkyl substituent at the nitrogen atom (methyl–butyl–octyl) in a series of **1**–**2**–**3** led to an increase in the number of preferred conformers from five for **1** to six for **2** and **3**. However, the structures of the corresponding preferred conformers differ slightly, and steric factors—a flat amide fragment, the size of bulky phenyl groups, and the configuration of alkyl bridges—are important.

## 3. Materials and Methods

### 3.1. Materials

*N*-methyl-*N*-[(2-(diphenylphosphoryl)ethyl]diphenylphosphorylamide **1**, *N*-butyl-*N*-[(2-(diphenylphosphoryl)ethyl] diphenylphosphorylacetamide **2**, and *N*-octyl-*N*-[(2-(diphenylphosphoryl)ethyl] diphenylphosphorylacetamide **3** were synthesized according to the following procedure [37].

### 3.2. IR Spectroscopy

The infrared spectra of crystals were collected on a FTIR Bruker Vertex 70 spectrometer (Bruker, Ettlingen, Germany) (600–4000 cm^−1^) with a single reflection, germanium crystal ATR accessory (MIRacle, PIKE Technologies) purged under dry air to remove atmospheric water vapor. The interferograms were recorded with a resolution of 2 cm^−1^, 128 scans, and Fourier transformed using a Blackman–Harris apodization function. The thin films of molten compounds were produced by the heating of crystal between KBr plates in home-made electrical oven. Temperature was measured by the PT100 sensor and was kept constant using a PID controller to ensure a standard deviation smaller than 1 K. The solid phase of samples was produced by slow cooling of thin film of molten compound (liquid) between KBr plates. The crystallization of the films was observed visually between crossed polarizers. The comparison spectra of the solid phases after melting and followed crystallization and the crystals of the same compounds showed their identity. Thus, there was no decomposition of the samples studied upon melting. KBr cells were used with a spacer (0.2 mm) to achieve the best signal/noise ratio. Concentrations of compounds were varied from 0.05 to 0.1 mol/L. Chloroform purified using molecular sieves was used as a solvent.

### 3.3. Dipole Moments

The experimental values of the dipole moments were determined using the second Debye method [38]. Physical parameters of **1**–**3** were measured for series consisting of 4–6 solutions in benzene at 25 °C. The dielectric permittivities of solutions of **1**–**3** were determined on a BI-870 instrument (Brookhaven Instruments Corporation, New York, USA); the accuracy is ±0.01. The refractive indices of solutions were measured on a RA-500 refractometer (Kyoto Electronics, Kyoto, Japan); the accuracy is ±0.0001.

The experimental dipole moments were calculated by the Debye Equation (1) [38]:

(1) $$\mu =0.01283\sqrt{P_{\mathrm{or}.}T}.$$

The orientation polarizabilities *P*_or_. were calculated by the Guggenheim-Smith Equation (2) [43,44]:

(2) $$P_{\mathrm{or}.}=\frac{M}{d}\left[\frac{3\alpha }{{\left(\varepsilon_{0}+2\right)}^{2}}-\frac{3\gamma }{{\left(n_{0}^{2}+2\right)}^{2}}\right]$$

where *M* is the molecular weight of a substance, *d* is the solvent density, *α* and *γ* are slopes of the *ε_i_*–*w_i_* and $n_{i}^{2}$–*w_i_* plots; *ε_i_*, *n_i_*, and *w_i_* are the dielectric constant, refractive index, and mass fraction of the solute of the *i*th solution, respectively. Equations for *α* and *γ* and the *ε_i_–w_i_* and $n_{i}^{2}$–*w_i_* plots (Figures S4–S6) are given in Supplementary Materials.

In the calculations of dipole moments according to the vector-additive scheme, we used the theoretical geometry parameters and following dipole moments of bonds and groups: *m*(C_Ph_→P) 1.09 D, calculated from μ_exp_ (C_6_H_5_)_3_P [41]; *m*(C=>O) 1.94 D [45]; *m*(P=>O) 2.94 D, calculated from μ_exp_ C_6_H_5_P=O [41]; *m*(C_sp3_→P) 0.83 D [41]; *m*(C_sp3_→C_sp2_) 0.75 D, calculated from μ_exp_ C_6_H_5_CH_3_ [38]; *m*(N→C_sp2_) 0.94 D, calculated from μ_exp_ CH_3_C(O)NH_2_ [38]; *m*(H→C_sp3_) 0.28 D [46].

### 3.4. Quantum Chemical Calculations

Quantum chemical calculations with full geometry optimization were performed using the density functional theory method B3PW91 [47,48] and the 6-311++G(df,p) [49] extended basis set (calculations of the molecules in vacuum) using a GAUSSIAN 09 software package [50]. In all cases, the geometric parameters of the molecules were fully optimized. The solvent has not been taken into account. Correspondence of the found stationary points to the energy minimums was confirmed by calculation of the second derivatives of energy with respect to the atom coordinates. All structures identified as energy minima were characterized by Hessians containing only positive frequencies.

## 4. Conclusions

We have determined the polarities and carried out experimental and theoretical conformational analysis of *N*-methyl-*N*-[2-(diphenylphosphoryl)ethyl]diphenylphosphorylacetamide, *N*-butyl-*N*-[2-(diphenylphosphoryl)ethyl]diphenylphosphorylacetamide, and *N*-octyl-*N*-[2-(diphenylphosphoryl)ethyl]diphenylphosphorylacetamide by the methods of dipole moments, IR spectroscopy, and DFT calculations.

In solution, compounds **1**–**3** exist as a conformational equilibrium of several forms with a predominance of highly polar rotamers. Preferred conformers **1a**–**e**, **2a**–**f**, and **3a**–**f** are divided into two groups in which the carbonyl group and alkyl substituent have *Z*- or *E*-configuration, and the phosphoryl-containing fragments are *syn*- or *anti*-located relative to the amide plane. The substituents at the phosphorus atoms have eclipsed *cis*- or staggered *gauche*-orientation relative to the phosphoryl groups, and the eclipsed *cis*-orientation of the substituents is due to the presence of intramolecular hydrogen bonds P = O⋯H–C_phenyl_ or p,π-conjugation between the phosphoryl group and the phenyl ring. Additional stabilization of preferred conformers of **1**–**3** is provided by various intramolecular hydrogen contacts with the participation of oxygen atoms of the phosphoryl or carbonyl groups and hydrogen atoms of the methylene and ethylene bridges, alkyl substituents, and phenyl rings. However, steric factors, such as a flat amide fragment, the size of bulky phenyl groups, and the configuration of alkyl bridges, make a significant contribution to the realization of preferred conformers.

The studied tridental phosphorylated acetamides are prospective extractants of various metal ions and ligands for the preparation of metal complexes with catalytic and biological activity. The results of the present study can be useful for the prediction and rationalization of properties and reactivity of modified carbamoylphosphine oxides.

## Figures and Tables

**Table 1:** Coefficients of calculated equations (*α*, *γ*), orientation polarizabilities (*P*_or_.), and experimental dipole moments (μ) of compounds **1**–**3** in benzene.

Compound	*α*	*γ*	*P*_or_., cm^3^	μ, D
**1**	6.106	0.303	547.82	5.15
**2**	5.070	0.214	496.84	4.90
**3**	4.091	0.259	432.32	4.77

**Table 2:** Relative energies (Δ*E)*, Gibbs free energies (Δ*G*, 298.15 K*)* (kJ/mol), theoretical (μ_theor)_ and calculated by the vector-additive scheme (μ_calc)_) dipole moments (D), percentage (*n*) of preferred conformers of **1**–**3** according to DFT B3PW91/6-311++G(df,p).

Conformer		Δ*E*	Δ*G*	μ_theor_	μ_calc_	*n*, %
**1a**	*Z*, *syn*	0.0	0.0	5.67	4.75	41.3
**1b**	*E*, *syn*	1.5	0.4	5.44	5.15	35.3
**1c**	*Z*, *syn*	2.9	8.2	1.72	1.77	1.5
**1d**	*E*, *anti*	3.7	1.6	5.53	5.84	21.5
**1e**	*Z*, *anti*	5.6	11.5	3.74	3.40	0.4
**2a**	*Z*, *syn*	0.0	2.1	5.48	4.74	23
**2b**	*E*, *syn*	1.6	0.0	5.22	5.33	55
**2c**	*Z*, *syn*	3.0	9.8	2.35	2.05	1
**2d**	*Z*, *syn*	3.4	5.7	5.71	4.77	6
**2e**	*E*, *syn*	5.1	6.0	5.54	5.51	5
**2f**	*E*, *anti*	5.4	4.3	5.30	5.80	10
**3a**	*Z*, *syn*	0.0	1.8	5.44	4.75	21
**3b**	*E*, *syn*	1.5	0.0	5.18	5.43	43
**3c**	*Z*, *syn*	2.9	9.3	2.34	2.28	1
**3d**	*Z*, *syn*	3.4	5.0	5.69	4.32	6
**3e**	*E*, *syn*	5.0	3.8	5.55	5.44	9
**3f**	*E*, *anti*	5.3	2.0	5.27	5.80	20

**Table 3:** Selected dihedral angles (degree) for the preferred conformers of **1–3** according to DFT B3PW91/6-311++G(df,p): *α* O1 = P1–C1–C2, β O1 = P1–C3–C4, *γ* O1 = P1–C6–C7, δ P1–C6–C7 = O3, *ε* O3 = C7–N–C15, ζ C7–N–C15–C16, η C15–N–C8–C9_,_ θ N–C8–C9–P2, **ι** C8–C9–P2 = O2, κ O2 = P2–C10–C11, λ O2 = P2–C13–C14.

	*α*	β	*γ*	δ	*ε*	ζ	η	Θ	ι	κ	λ
**1a**	−1	22	82	93	0	-	−84	168	−65	−14	7
**1b**	6	24	75	99	−178	-	100	−179	60	8	31
**1c**	59	12	47	89	−2	-	−73	−166	41	72	5
**1d**	7	25	77	104	173	-	−94	178	−61	−30	−9
**1e**	61	15	71	100	3	-	80	134	−58	36	17
**2a**	−1	22	82	92	0	87	−84	167	−65	−15	7
**2b**	6	25	74	97	−179	−97	100	−178	60	8	31
**2c**	59	12	59	76	−3	−84	−92	−153	49	72	5
**2d**	−2	22	79	95	−1	−77	−79	168	−67	−15	7
**2e**	7	26	74	98	−179	−97	100	−179	60	9	31
**2f**	6	24	77	100	−179	−102	−109	179	−60	−30	−8
**3a**	−1	22	81	92	−1	87	−84	168	−65	−14	7
**3b**	6	25	74	97	−179	−96	100	−178	60	8	31
**3c**	58	13	60	76	−3	−84	−91	−153	49	70	5
**3d**	−1	23	79	95	−1	−77	−79	167	−68	−15	6
**3e**	8	26	75	103	179	121	108	−179	60	8	31
**3f**	6	25	77	100	−179	−101	−108	179	−60	−29	−7

**Table 4:** Selected geometry characteristics of hydrogen bonds (distance, Å; angle, degrees) according to DFT B3PW91/6-311++G(df,p).

	H5⋯O1	C9–H5⋯O1	H6⋯O1	C12–H6⋯O1
**1a**	2.194	153	2.339	164
**1b**	2.247 *	151 *		
**1c**	2.160	144	2.305	157
**1d**	2.236	152		
**1e**	2.175 **	152 **	2.422	154
**2a**	2.189	154	2.341	164
**2b**	2.271 *	153 *		
**2c**	2.152	162	2.255	172
**2d**	2.248	147	2.323	163
**2e**	2.259 *	153 *		
**2f**	2.189	154	2.350	163
**3a**	2.272 *	153 *		
**3b**	2.162	161	2.248	172
**3c**	2.250	147	2.323	163
**3d**	2.260 *	153 *		
**3f**	2.194	153	2.339	164

* H7⋯O1, C15–H7⋯O1; ** H4⋯O1, C8–H4⋯O1.

**Table 5:** Selected vibration frequencies of the phosphoryl and carbonyl groups of **1**–**3**; theoretical values are listed for conformers a–f.

	P=O			C=O		
	1	2	3	1	2	3
ν_exp_ solid	1179, 1188	1187	1181	1648	1642	1632
ν_exp_ melt	1194	1194	1195	1643	1640	1641
ν_exp_ solution	1184	1184	1182	1639	1639	1634
ν_theor_ a	1178, 1187	1177, 1182, 1194	1179, 1184, 1187	1650	1644	1644
ν_theor_ b	1187, 1192	1190, 1193	1188, 1192, 1193	1642	1639	1639
ν_theor_ c	1193	1162, 1175	1162, 1175	1641	1652	1652
ν_theor_ d	1171, 1181	1179, 1181, 1191	1179, 1184	1647	1643	1643
ν_theor_ e	1180, 1183	1190, 1194	1191, 1194	1659	1632	1632
ν_theor_ f	-	1190, 1193	1192, 1193	-	1635	1635

## Supplementary Materials

Figure S1: IR spectra of compound **2** in different aggregate states, Figure S2: IR spectra of compound **3** in different aggregate states, Figure S3. Visualization of the molecular orbitals for **1e** (the interaction between the P = O bond and phenyl substituents is absent). The positive and negative potentials are shown as blue and red areas, respectively, Equations for *α* and *γ* calculations (Guggenheim–Smith equation) and the *ε_i_*–*w_i_* and $n_{i}^{2}$–*w_i_* plots, Figure S4. The *ε_i_*–*w_i_* and $n_{i}^{2}$–*w_i_* plots for compound **1**. Figure S5. The *ε_i_*–*w_i_* and $n_{i}^{2}$–*w_i_* plots for compound **2**. Figure S6. The *ε_i_–w_i_* and $n_{i}^{2}$–*w_i_* plots for compound **3**.

## Sample Availability

Samples of the compounds **1**–**3** are not available from the authors.
