# Supplementary material for: Conformational Analysis of N-Alkyl-N-[2-(diphenylphosphoryl)ethyl]amides of Diphenylphosphorylacetic Acid: Dipole Moments, IR Spectroscopy, DFT Study
Source: Molecules. 2021 Aug 10;26(16):4832. doi: 10.3390/molecules26164832 (PMC8399383; doi:10.3390/molecules26164832)
Supplement: Supplementary file 1 [file molecules-26-04832-s001.zip › molecules-1309077-supplementary.pdf]

Supplementary material

Conformational Analysis of *N*-Alkyl-*N*-[2-(diphenylphosphoryl)ethyl]amides of Diphenylphosphorylacetic acid: Dipole Moments, IR spectroscopy, DFT Study

Anastasia Kuznetsova [1], Denis Chachkov [2], Oleg Artyushin [3], Natalia Bondarenko [4,5] and Yana Vereshchagina [1,*]

[1] Department of Physical Chemistry, A.M. Butlerov Institute of Chemistry, Kazan Federal University, Kremlevskaya 18, 420008 Kazan, Russia; kuznetsovaanastan@gmail.com
[2] Kazan Department of Joint Supercomputer Center of Russian Academy of Sciences–Branch of Federal Scientific Center"Scientific Research Institute for System Analysis of the RAS", Lobachevskogo 2/31, 420111 Kazan, Russia; de2005c@gmail.com
[3] A.N. Nesmeyanov Institute of Organoelement Compounds, Russian Academy of Sciences, Vavilova 28, 119991 Moscow, Russia; oleg.artyushin@gmail.com
[4] [a]Institute of Chemical Reagents and High Purity Chemical Substances of National Research Centre "Kurchatov Institute", Bogorodsky val 3, 107076 Moscow, Russia;, Moscow, Russia; bond039@mail.ru
* Correspondence: jveresch@kpfu.ru or yavereshchagina@yahoo.com

Contents

Figure S1. Visualization of the molecular orbital HOMO-29 (E = -10.617 eV) for 1c (the interaction between the P=O bond and phenyl substituents is absent). The positive and negative wave functions are shown as blue and red areas, respectively. Isocontour value is 0.02 a.u. — pS2
Figure S2. IR spectra of compound **2** in different aggregate states. D is the absorbance. — pS3
Figure S3. IR spectra of compound **3** in different aggregate states. D is the absorbance. — pS4
Equations for α and γ calculations (Guggenheim-Smith equation) — pS5
Figure S4. The ε_i–w_i and n_i^2–w_i plots for compound **1**. — pS6
Figure S5. The ε_i–w_i and n_i^2–w_i plots for compound **2**. — pS7
Figure S6. The ε_i–w_i and n_i^2–w_i plots for compound **3**. — pS8

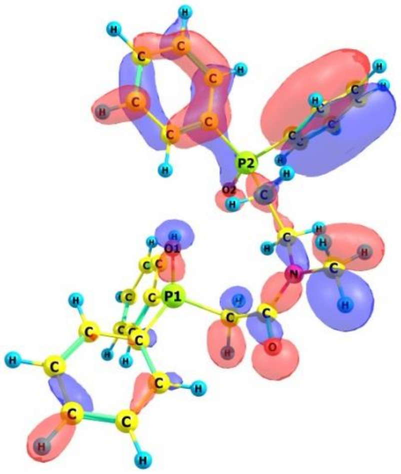

Figure S1. Visualization of the molecular orbital HOMO-29 (E = -10.617 eV) for **1c** (the interaction between the P=O bond and phenyl substituents is absent). The positive and negative wave functions are shown as blue and red areas, respectively. Isocontour value is 0.02 a.u.

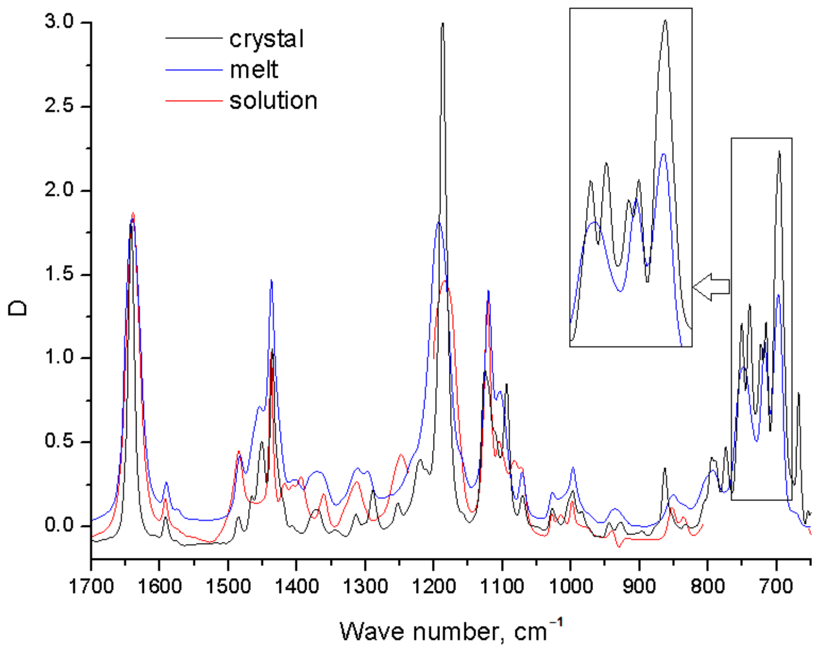

Figure S2. IR spectra of compound **2** in different aggregate states. D is the absorbance.

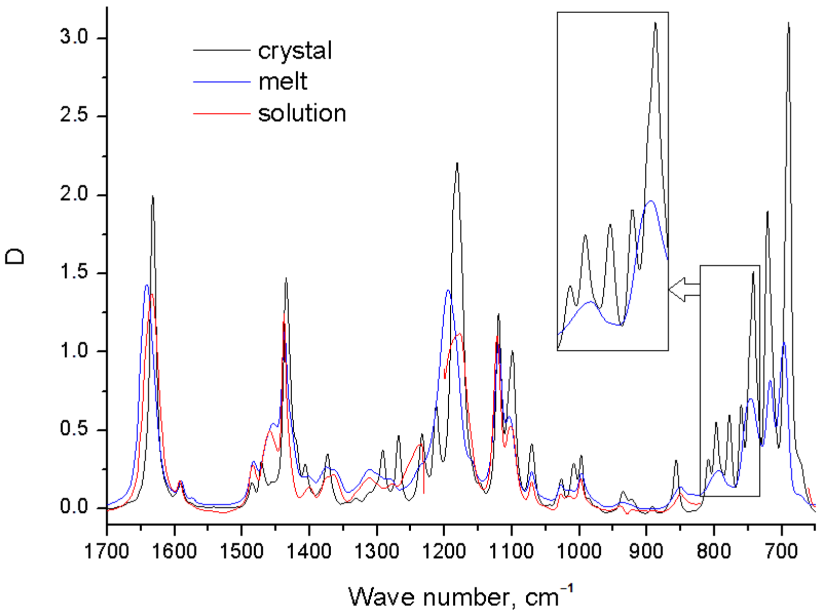

Figure S3. IR spectra of compound **3** in different aggregate states. D is the absorbance.

Equations for α and γ calculations (Guggenheim-Smith equation):

$$\alpha = \frac{\varepsilon_i - \varepsilon_0}{\omega_i}$$

$$\gamma = \frac{n_i^2 - n_0^2}{\omega_i}$$

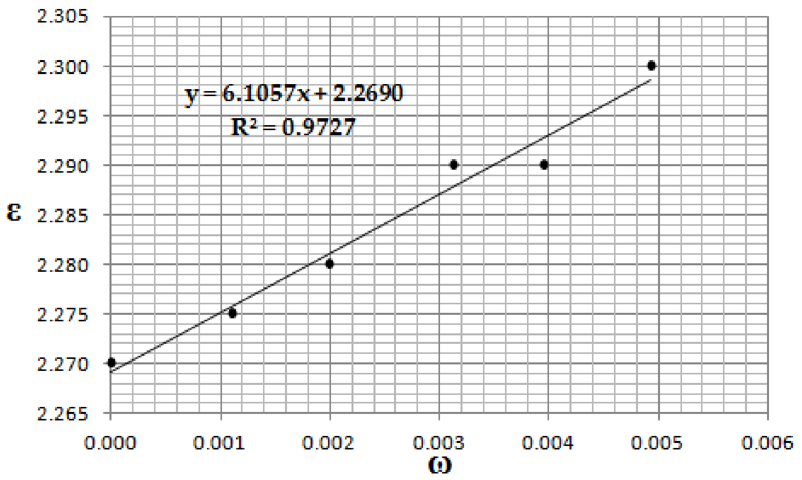

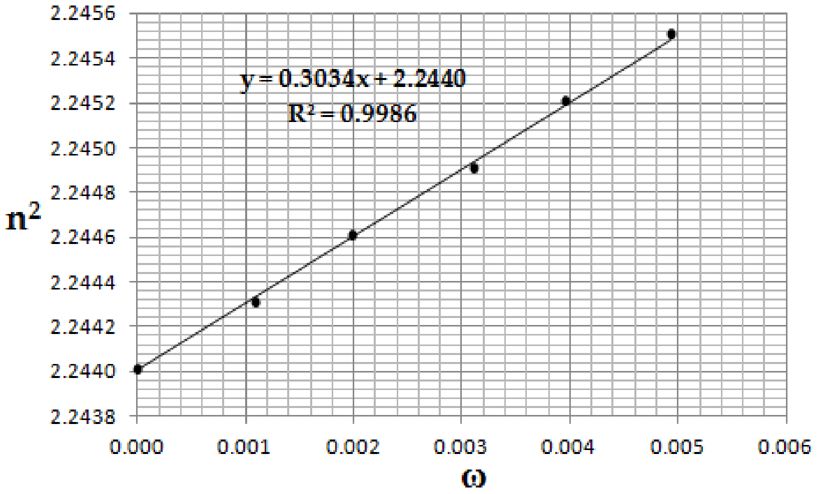

Figure S4. The ε_i–w_i and n_i^2–w_i plots for compound **1**.

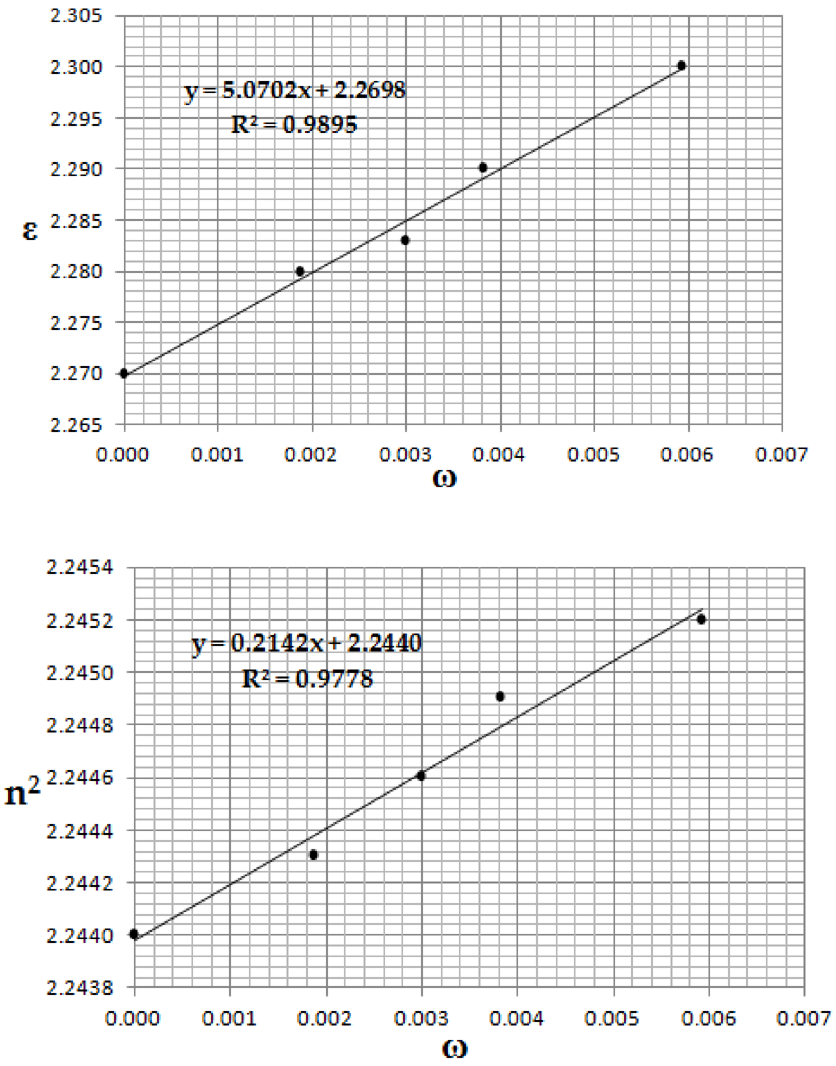

Figure S5. The ε_i–w_i and n_i^2–w_i plots for compound **2**.

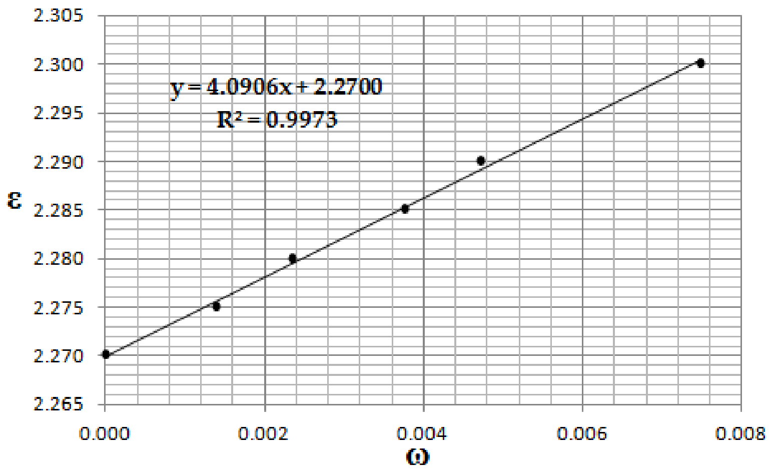

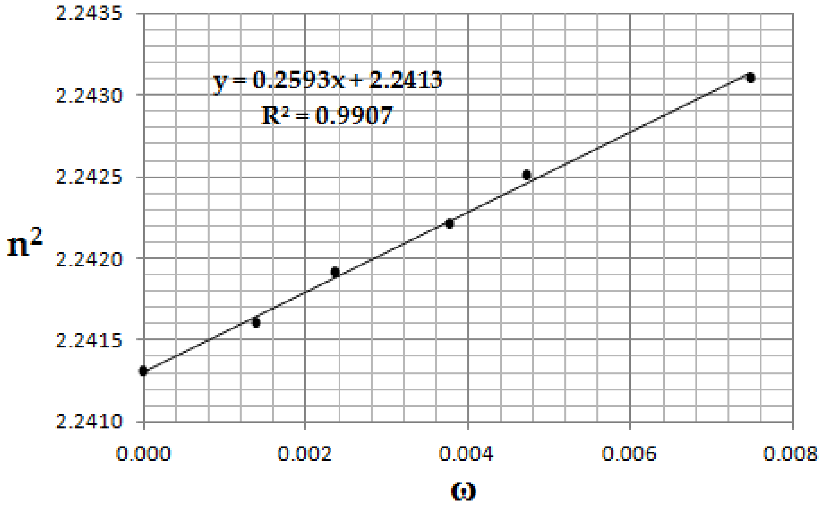

Figure S6. The ε_i–w_i and n_i^2–w_i plots for compound **3**.
